# Design and Implementation of an Urban Farming Robot

**DOI:** 10.3390/mi13020250

**Published:** 2022-02-02

**Authors:** Michail Moraitis, Konstantinos Vaiopoulos, Athanasios T. Balafoutis

**Affiliations:** Institute of Bio-Economy & Agro-Technology, Centre of Research & Technology Hellas, Dimarchou Georgiadou 118, 38333 Volos, Greece; k.vaiopoulos@certh.gr (K.V.); a.balafoutis@certh.gr (A.T.B.)

**Keywords:** urban agriculture, robot, vegetables, plant detection, precision irrigation

## Abstract

Urban agriculture can be shortly defined as the growing of plants and/or the livestock husbandry in and around cities. Although it has been a common occupation for the urban population all along, recently there is a growing interest in it both from public bodies and researchers, as well as from ordinary citizens who want to engage in self-cultivation. The modern citizen, though, will hardly find the free time to grow his own vegetables as it is a process that requires, in addition to knowledge and disposition, consistency. Given the above considerations, the purpose of this work was to develop an economic robotic system for the automatic monitoring and management of an urban garden. The robotic system was designed and built entirely from scratch. It had to have suitable dimensions so that it could be placed in a balcony or a terrace, and be able to scout vegetables from planting to harvest and primarily conduct precision irrigation based on the growth stage of each plant. Fertigation and weed control will also follow. For its development, a number of technologies were combined, such as Cartesian robots’ motion, machine vision, deep learning for the identification and detection of plants, irrigation dosage and scheduling based on plants’ growth stage, and cloud storage. The complete process of software and hardware development to a robust robotic platform is described in detail in the respective sections. The experimental procedure was performed for lettuce plants, with the robotic system providing precise movement of its actuator and applying precision irrigation based on the specific needs of the plants.

## 1. Introduction

Nowadays, more than 50% of the world population lives in cities and by 2030 this percentage will reach 80% [1]. The urbanization process has led food availability to extensive supply chains that in most cases end-up at super markets [2]. Urban agriculture (UA) and peri-urban agriculture can be defined as the growing, processing, and distribution of food and other products through plant cultivation and seldom raising livestock in and around cities for feeding local populations; and has important broad benefits for the citizens worldwide [3], such as (i) Shortened supply chains, (ii) Carbon sequestration, (iii) Potentially reduced urban heat, (iv) Improved physical and mental health, (v) Improved aesthetics, (vi) Community building, (vii) Employment opportunities, (viii) Improved local land prices, (ix) Provision of habitats for wildlife, (x) Waste recycling. Thus, UA can be seen not only as a source of fresh food, but also as a mechanism of social integration, economic development and environmental sustainability [4]. Especially in terms of economics, rooftop gardens in cities could supply up to 77% of the vegetables consumed in them, if all suitable flat roof spaces across them were utilized accordingly [5]. Even in times of crisis, UA played a major role in food production [6,7]. Other studies indicate the potential to attribute “significant economic returns” by utilizing cities’ green spaces to execute urban agriculture at 25% to 50% of their area, based on estimations of horticultural crops’ yields [7].

UA has found application mainly in developing countries, as a means of family food security and income provider [8,9,10]. However, in developed countries, time availability is the main problem hindering adoption of this system, as medium to high-income professionals do seek high quality homemade fresh horticultural products, but do not have the time to take care of a family garden appropriately. Therefore, development of automated solutions could contribute in solving this problem.

Different technologies and automations such as simple integrated circuits to more complex microcontrollers, sensors, micro-computers and Internet of Things (IoT) applications [11,12,13]; have been implemented in agriculture through smart farming applications (SFT) that promote (i) data acquisition, (ii) data analysis-evaluation and (iii) precision application [14], with significant positive impacts in environmental, economic and labor parameters [15,16]. However, these applications are present in large-scale farming, while very limited work has been done in small-scale, automated UA.

Data acquisition tasks are supported by the growing computer vision techniques and access to multiple sensory data [17,18,19,20,21], while data analysis and evaluation tasks are becoming more and more common in agricultural research [22,23,24]. A combination of data acquisition with data analysis and evaluation is also done in the continuously developing domain of object detection based on convolutional neural networks (CNNs). Many different algorithms have been developed and further optimized with successful application examples dealing with leaf disease detection [25], plant disease detection [26], land cover classification [27], crop type classification [28], plant recognition [29], crop yield estimation [30], fruit counting [31], weed management [32] and many more [33].

The precision in application as part of SFTs is provided by various types of robotic systems that have been developed and are being utilized depending on the application. Unmanned Ground Vehicles (UGV), Unmanned Aerial Vehicles (UAVs) and robotic arms are already used in multiple agricultural activities [34,35]. Cartesian robots on the other hand are typically used in 3D printing and CNC applications [36,37,38]. Inspired by the precision in movement that Cartesian robots can achieve in combination with their adaptable size and ease of application in urban environments (backyards, balconies, rooftops, etc.), the objective of this work was to design, assemble and test an automated Cartesian robotic system for provision of fresh vegetables to city dwellers in situ.

Except this introductory part, this paper presents in Section 2 the materials used to construct the robotic system (hardware) and the software developed for operating it, together with the experimental apparatus and the methodology followed to assess the system’s accuracy. In addition, Section 3 exhibits the results and the respective discussion over the conducted experiments of the three main aspects of the robotic system; namely image stitching, plant detection-localization, and movement precision. Finally Section 4 concludes this work and describes the future directions for research.

## 2. Materials and Methods

A 3D robotic platform named CityVeg has been developed for the automatic monitoring and management of urban gardens. The implementation is divided into (a) the hardware, presenting the design and construction of CityVeg and (b) the software, describing the system pipeline that feeds CityVeg with the commands to be executed.

### 2.1. Hardware—Design and Construction

For the design and construction of CityVeg, factors such as the cost and the availability of materials and tools, the ease of assembly and the maintenance costs were considered. Moreover, a number of prerequisites were defined early in the process, namely:

- The system as a whole has to be light, robust and resilient in respect to external factors such as weather conditions;
- The system should be able to cover the size of a small urban garden, e.g., for being placed in a balcony, but also easily scalable if needed;
- In order for the vertical growth of plants to be also taken into account, the irrigation nozzle (actuator) should have 3 Degrees of Freedom (DoF) to allow three-dimensional movement;
- The actuator should move with the utmost precision to reach accurately each of the respective plants;
- Its components should not impede plant growth and;
- No human intervention should be necessary at any stage of the plant development.

The full list of the parts; either designed and 3D printed, or bought; can be found in Table A1 of Appendix A.

#### 2.1.1. Frame and Moving Parts

A 3D printer layout was selected to achieve precise 3-axis movement. The aluminium frame consists of 2 parallel horizontal rails, a separate aluminium frame with a pi (Π) shape that runs on the rails and the actuation component that runs horizontally and vertically, mounted on the Π frame, thus achieving 3 DoF for the actuator at its lowest point.

**Figure 1:**
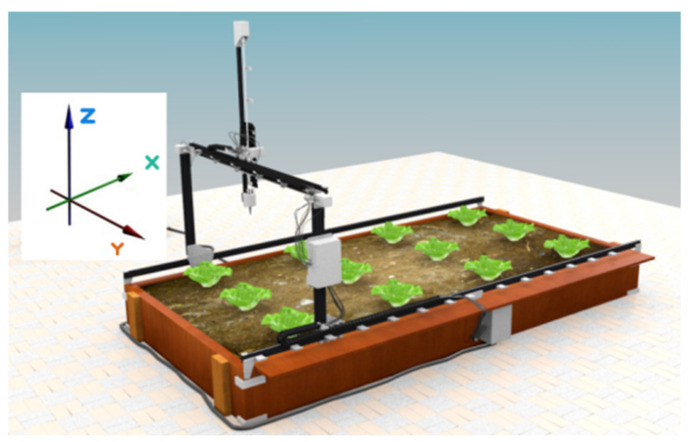
Plain schematic of CityVeg’s layout.

For easier understanding of the layout, 3 axes of movement are defined (Figure 1):

- X axis—movement of Π frame across the 2 parallel horizontal rails;
- Y axis—movement of the vertical bar across the upper part of the Π frame;
- Z axis—vertical movement of the vertical bar which bears the actuator.

**Figure 2:**
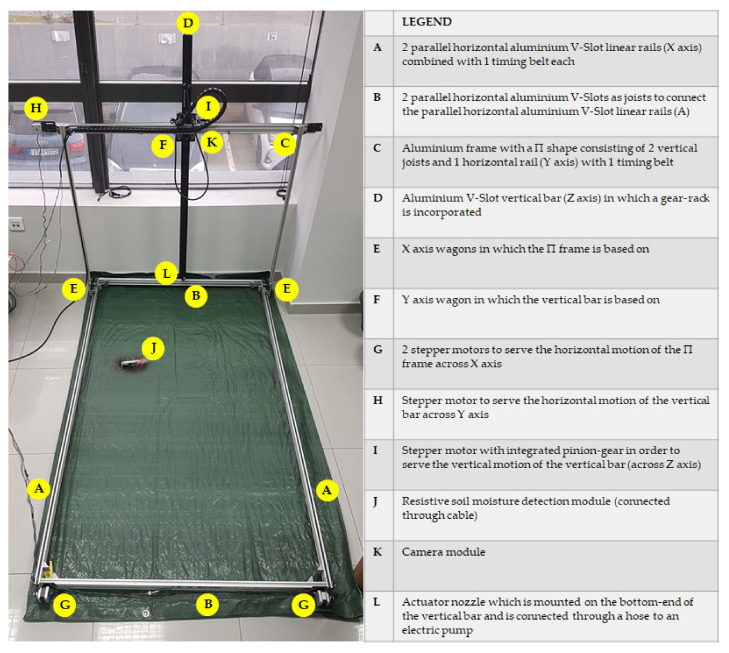
View of the assembled CityVeg platform, indicating the position of each specific part.

**Figure 3:**
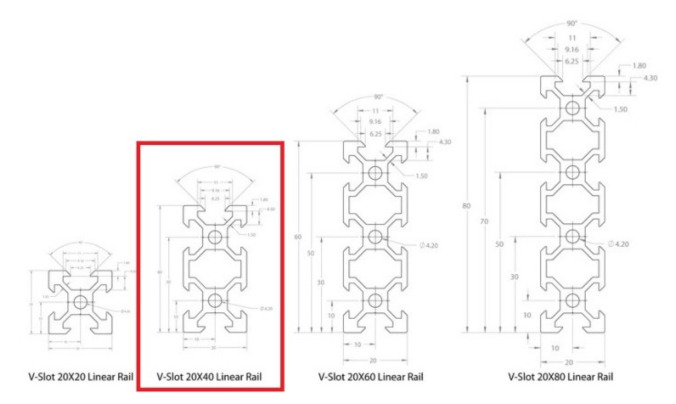
V-Slot aluminum profiles’ types and dimensions.

The assembled platform is shown on Figure 2. More specifically, the whole CityVeg frame was assembled mainly by combining aluminium V-Slot linear rails (20 mm × 40 mm). In general, V-Slot extrusions are high quality linear aluminium profiles with V-shaped internal channels on all 4 sides, which allow the linear movement of the respective V-Slot wheels (Figure 3) [39]. The frame’s final dimensions are 1.75 m × 1 m × 1 m, based on the prerequisites of Section 2.1. The vertical bar has a length of 1.25 m, slightly longer than the height of the Π frame, so to be able to descend to ground level and apply irrigation from the lowest possible height in the early stages of plant growth.

**Figure 4:**
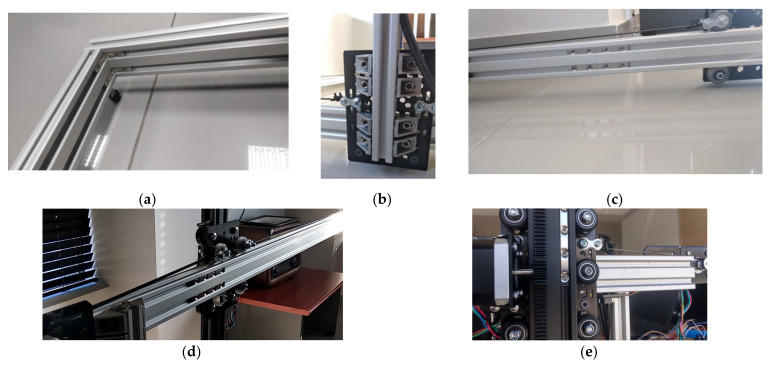
Details of the: (**a**) Frame’s inside hidden corner brackets; (**b**) V-Slot wagon and 90-degree corner brackets holding the Π frame (**c**) Frame’s quad connecting Tee Nuts for end to end V-Slot connections; (**d**) X axis layout and; (**e**) Z axis wagon and motor layout.

The smooth movements of (a) the Π frame on the 2 parallel horizontal rails and (b) the vertical actuation component on the upper (horizontal) rail of the Π frame are achieved by utilizing the properly adapted V-Slot plates and wheels. The aforementioned combination will be called from this point forward “wagons”. The plates are made of anodized aluminium alloy 6036-T6 (127 mm × 88 mm × 3 mm) and the wheels are made of polycarbonate plastic (outer diameter of 24.39 mm, pressure limits up to 86 Mpa). The stability of the frame is ensured by the respective joints between extrusions and eccentric spacers between wheels of opposite rail sides (Figure 4).

#### 2.1.2. Motors and Transmission

**Figure 5:**
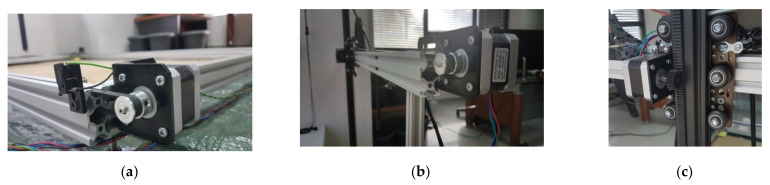
Detailed view of the motors of: (**a**) X axis (1 of the 2); (**b**) Y axis and; (**c**) Z axis.

The movement of individual parts of CityVeg is performed by 4 stepper motors (12 V, 0.4 A/phase, NEMA17, 200 steps/rev, T = 28 N.cm by Wantai Motors, Changzhou, China). Two of these motors are utilized for the uniform movement of the Π frame onto the 2 parallel horizontal rails, also combined with 2 idler pulleys at the opposite side of the rails and 2 GT2-type (2 mm Pitch) timing belts connected to the wagons that bear the Π frame. The third wagon movement along the horizontal rail of the Π frame was arranged similarly (3rd motor). All 3 timing belts run partly inside the hollow of the aluminium profiles (Figure 5).

**Figure 6:**
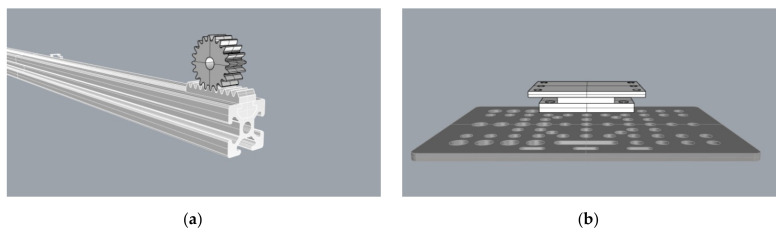
(**a**) Gear/rack graphic designs on the V-Slot extrusion and; (**b**) Z axis motor base design on the V-Slot wagon.

The 4th motor is utilized for the vertical movement of the vertical bar which bears the actuator. A rack was mounted along the vertical bar and a gear was fitted to the 4th motor. Thus, the vertical bar does not impede plant growth, therefore fulfilling the relevant prerequisite of Section 2.1. This is also the reason the common “screw driven” layout was not selected. The gear and the rack (Mod.1—15 mm × 15 mm) were designed and 3D-printed together with the motor base (Figure 6). The final arrangement of the Z axis wagon is shown in Figure 4e. The motors’ connection and control was achieved by 4 stepper drivers A4988 [40] coupled to the motors, Arduino and power supply.

#### 2.1.3. Sensors and Switches

**Figure 7:**
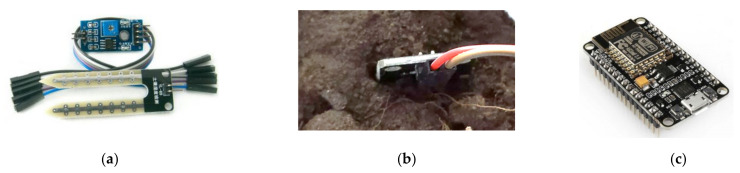
(**a**,**b**) Soil humidity sensor and; (**c**) NodeMCU microcontroller.

For the monitoring of the urban garden, a soil hygrometer (soil moisture sensor) and an RGB camera are utilized. The purpose of humidity monitoring is to evaluate soil water content before each application cycle, in case sufficient water was provided by external factors, such as rain. An economic resistive soil moisture detection module has been installed (Figure 7a,b). The provision of the analogue signal which indicates the current soil moisture was utilized. The threshold value for the need of irrigation was empirically set at 95% soil moisture in order for the irrigation application to be avoided in rainy days. For cloud communication the NodeMCU (a Lua based ESP8266) [41] was selected, also providing scalability in case it is deemed appropriate to add more sensors of any type in the future (Figure 7c).

**Figure 8:**
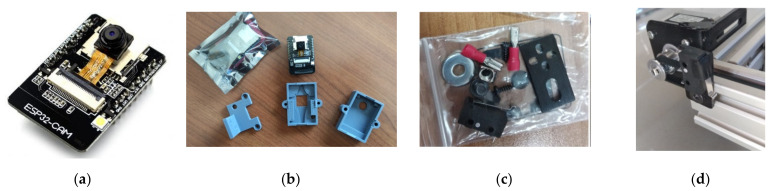
(**a**) ESP-32 CAM; (**b**) Camera, camera casing design and 3D printed casing; (**c**) Limit switch components and; (**d**) Installed limit switch.

The second input of the system is a camera that sends multiple images of the garden parcel to the cloud before each application. The purpose of taking multiple photos lays in the quality of the final combined image which will result by the unification of the different images and will be fed into the neural network at a next step (Section 2.2.2). The selected camera module is the ESP32-CAM, a compact, low-power OV2640 camera based on ESP32 that offers among else a resolution of 1600 × 1200 pixels, auto-focus and built-in Wi-Fi/Bluetooth modules (Figure 8a) [42] and is suitable for small-scale IoT solutions, in terms of cost, performance and energy consumption [43]. A plastic casing was designed and 3D printed aiming to protect it from external factors and mount it on the frame (Figure 8b). It was placed on the third wagon as this position offers multiple advantages, namely:

- Fixed height with sufficient field of view across the width of the parcel;
- Ability to move along the X axis to take multiple photos for a complete depiction of the garden;
- Unhindered view to the garden parcel provided that the actuation component (Z axis) rises above the camera lens;
- No need to install electronic parts onto the actuation component and;
- It is a protected position in general.

As the stepper motors do not return feedback regarding the reached position, 8 “limit switches” were installed to fully cover the ends of all axes (4 in X axis, 2 in Y and 2 in Z axis) for safety reasons and for creating a reference point/position (Figure 8c,d) [44].

#### 2.1.4. Irrigation System

**Figure 9:**
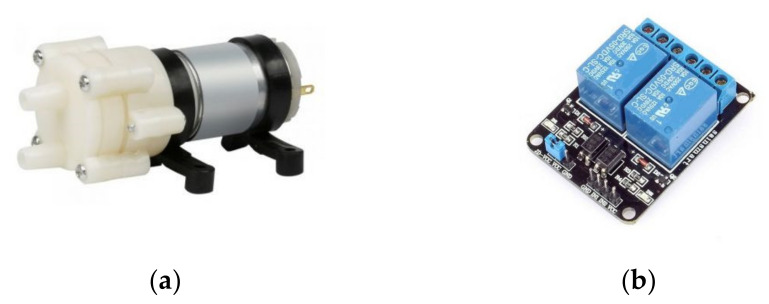
(**a**) Electric pump and; (**b**) Relay module.

To apply irrigation, a centrifugal electric pump (12 V DC, 0.67 A, 3 L/min maximum flow rate) was used together with a 20 L water tank. A 6 mm PE (Polyethylene) hose was used to channel the water up to the dripper of the actuation component. The control of the pump was achieved with a typical relay module connected to the Arduino (Figure 9).

#### 2.1.5. Arduino Microcontroller

In order to control the stepper motor drivers, the limit switches and the water pump relay, the Arduino Mega 2560 Rev3 was used [45]. Limit switches are very sensitive to noise, so to mitigate the disturbance, a 4.7 K resistor and a 100 nF capacitor were added in the wiring of each switch (in the 5 V and the GND output respectively). A Wi-Fi connection to the cloud was established via the ESP8266 Wi-Fi module ESP-12 [46]. Finally, a 12 V DC, 8.5 A (102 Watt) power supply and the appropriate DC/DC voltage reducers were used to power all the components of the robotic system.

### 2.2. Software—System Pipeline

CityVeg is scheduled to be activated every 24 h and perform a sequence of processes—referred to from this point forward as “application cycle”. The cycle begins with the monitoring of the urban garden and ends with the application of precision irrigation. In short, the system every 24 h:

- Receives and sends the data from both sensors, meaning soil moisture and images, to the cloud;
- Processes the images before feeding them to the neural network;
- Identifies the plants exact position and classifies them according to their size and;
- Produces and forwards to the actuator the commands for the application.

**Figure 10:**
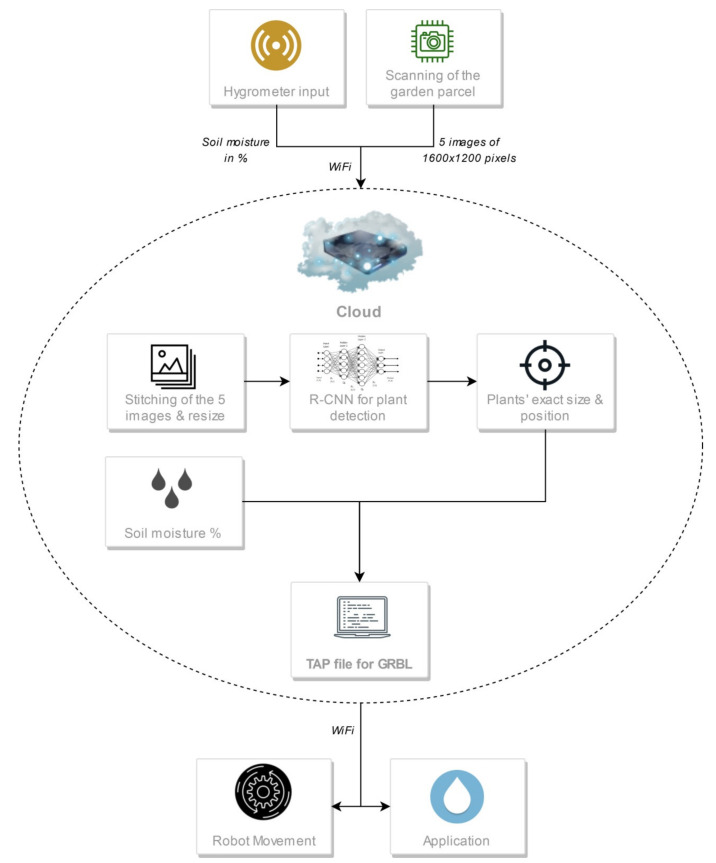
Flow chart of CityVeg overall system pipeline.

Figure 10 shows the flow chart of CityVeg overall system pipeline. 

#### 2.2.1. Urban Garden Monitoring and Image Acquisition

The initial process that the system must perform is the supervision of the urban garden. Regarding soil moisture, in this step it is considered adequate to send to the cloud the (%) percentage of soil moisture which will be utilized in the next step to assess the need to apply irrigation to the garden parcel.

**Figure 11:**
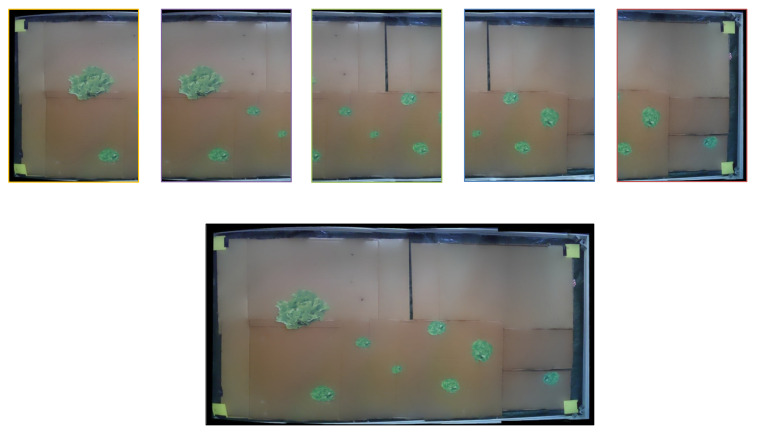
Image stitching example. Five separate images being combined into a single one.

The second input is the garden’s images. The images are captured in such a way to partially overlap each other so that during their unification in the next step, they have enough common sections for the process to be carried out and the photos to be successfully combined into a single, final image—panorama (Figure 11). This process is called image stitching or image mosaicking and the exact methodology that was followed is described in Section 2.2.2.

As shown in Figure 11, five (5) overlapping photos are combined into one. The process of image capturing consists of: (a) partial raise of the vertical component so as not to interfere with the camera’s field of view, (b) movement of the third wagon (Y axis) so that the camera’s lens is positioned in the middle of the Y axis and its field of view captures the full system’s width and (c) movement of the Π frame along the garden, consecutively stopping at the 5 fixed points where the photos are to be captured. Before each capture the Π frame is immobilized for 1.5 sec in order to dampen any oscillations that would lead to blurred images. Due to memory limitations each photo is captured and sent to the cloud before capturing the next one. All the commands above are given for predefined coordinates. When all 5 captures are completed, all axes return to the home position (X0, Y0, Z0). Details of the software that controls the movement are given in Section 2.2.4. 

#### 2.2.2. Data Processing

The purpose of data processing in the current system is: (a) the identification of the exact position of the plants, (b) their classification in 5 classes according to their size, (c) the determination of the soil moisture content threshold, below which irrigation should be applied, and (d) the determination of the irrigation dose for each individual plant.

The first step towards extracting plants’ position and size is the image stitching process. Algorithms for aligning images and combining them into a single image-mosaic are commonly used in Computer Vision; typical examples are the panorama mode in most commercial digital cameras and the creation of maps by combining multiple satellite or other aerial photos [47]. In the current system this process is done mainly by using the open library OpenCV 4.2 [48] in Python 3.5.6. OpenCV is an open source computer vision and machine learning software library, built to provide a common infrastructure for computer vision applications and to accelerate the use of machine perception in commercial products [49].

**Figure 12:**
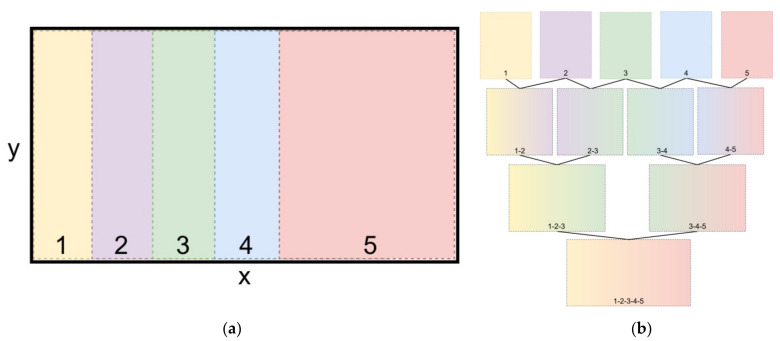
(**a**) Garden parcel segmentation for the 5 images’ acquisition and; (**b**) Image stitching process.

The field of view in each of the 5 captures in relation to the total area of the garden is shown graphically in Figure 12a. Each photo is captured with the same resolution (1600 × 1200 pixels) and covers the same area size, as the 5th capture. Due to overlap, sections of captures 1, 2, 3 and 4 are not shown in the figure. The image stitching process is graphically explained in Figure 12b. In the first step, each photo is combined with the one/two next to it. In the second step the stitched images—results of the first step—are being combined in pairs and in the third step the final, single image is produced. The above methodology emerged after multiple tests that resulted in this reliable application of the algorithm, mostly due to the high degree of overlap between the images that are going to be stitched, in each step of the process.

Having completed the stitching process, the produced single image depicts the total area of the garden parcel. The next step is its transformation so that its four sides become tangent in the inner borders of the frame that surrounds the garden and its dimensions correspond to its real dimensions. The transformation is carried out with the tool “cv2.getPerspectiveTransform” from the OpenCV library, in predefined dimensions that resulted from the accurate measurement and matching of the final image’s pixels to the real world.

Having defined its dimensions, the image is being fed into a neural network to extract the exact position and size of the plants. The open-source software library for machine learning by Google, TensorFlow [50,51] (GPU-enabled version) was utilized with a NVIDIA GeForce GTX 1060 Max-Q 6GB GPU. In order to make use of the specific GPU for the training, the parallel computing platform and application programming interface model by NVIDIA, CUDA (Compute Unified Device Architecture) [52] was used together with the library cuDNN (CUDA Deep Neural Network library) [53]. For compatibility reasons, specifically CUDA 10.0, cuDNN 7.4, and TensorFlow-GPU 1.13.2 were used in Python 3.5.6. “TensorFlow 1 Detection Model Zoo” provides several pre-trained detection models with specific neural network architectures. Some models, such as the SSD-MobileNet [54], use an architecture that allows faster detection but with less accuracy, while other models, such as the Faster-RCNN, provide slower detection but with greater accuracy. In general, Faster R-CNN [55] is a state of the art object detection model. It is a Region-Based Convolutional Neural Network (CNN) that is a single-stage model and can be trained end-to-end. It has integrated into its CNN a novel Region Proposal Network (RPN) which is the component that generates regions of interest for the system. As detection speed is not of major concern in the specific robotic system, the training was conducted on the Faster-RCNN-Inception-V2 model which is proved on the COCO (Common Objects in Context) dataset [56]. When a model is initially trained on a generic dataset, like COCO, it becomes able to learn well to identify both basic patterns and textures that tend also to appear in plenty of datasets. The result is that good generalization is achieved. The followed strategy is to construct a robust model that is going to be able to adapt quickly and easily to any case-specific dataset.

**Figure 13:**
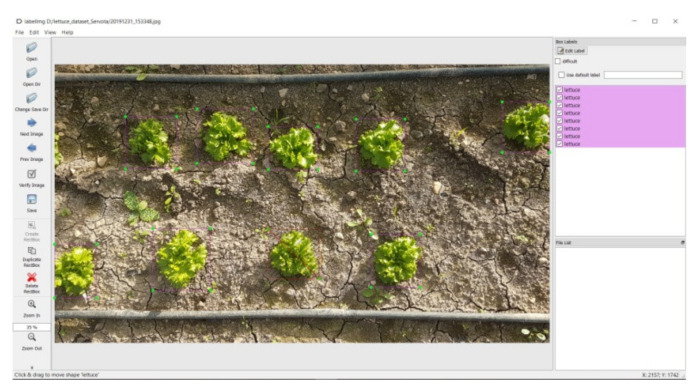
LabelImg tool and lettuce plant labeling.

For CityVeg development, a lettuce plant dataset was created from scratch. In order to create the dataset, 400 RGB images of lettuce plants at different stages of development (102 images of plants at early growth stage and 298 at advanced growth stage) were captured from open-field cultivations in Karditsa, Greece, under constant weather conditions. More specifically, all images were captured under natural lighting conditions. The weather during the time period of the day that the images were captured was sunny (approx. 100,000 lux, while the average monthly sunshine duration for the area is 109.1 h), at 8 °C and an average wind speed of 2.5 km/h (direction: W). Each image contained 8 to 22 plants and so, approximately 6000 lettuce plants were labelled. All camera shots were taken from 1 m height, so that the display of each plant is similar to the top view captures of the system’s camera. Training was conducted using 80% of the captured images (240 images with plants in advanced growth stage and 80 in early), while the remaining 20% (58 images with plants in advanced growth stage and 22 in early) was used for the testing procedure. The labelling process was carried out with a tool named LabelImg (Figure 13) [57,58].

The trained model was tested and was able to identify lettuce plants with an accuracy of up to 92%, which refers on accuracy on testing. For each detected lettuce plant the following data is extracted: (a) area of the bounding box that encloses it, (b) coordinates of the box’s centre point and (c) detection score having set a threshold of 80%. Anything detected with a lower score is not going to be irrigated. The data is exported in a CSV format and as a final step the entries are sorted based on their centres’ ascending X coordinate. The sorting serves the smooth movement of the actuation component during the application, as it will be picking the plants to be irrigated in an ascending X axis order, which is not the ideal (shortest) path but it is adequately short.

As shown in the flowchart (Figure 10), the soil moisture percentage is combined with the Faster-RCNN neural network’s input to produce the commands for the actions of CityVeg. In fact, a soil moisture threshold has been set at 95% as described above, above which the irrigation of the garden is considered unnecessary. The determination of this threshold was done in a first stage empirically, taking into account parameters such as the sensor’s depth, ground water availability and plant type, and was later experimentally corrected. Indicatively, most lettuce varieties as soon as they are transplanted have a root zone depth of 3–4 cm, while when the harvest season is approaching their roots have developed to a depth of up to 30 cm [59,60,61].

The precision irrigation concept that CityVeg aims to achieve consists of 4 main aspects [62]. The first concerns the application of the irrigation on the exact plant’s position and is met by the actuator’s precise movement. The second and third concern the irrigation dose and method of application and are explained in the next paragraph. The fourth concerns the timing of the application, meaning that no-application in the specific 24-h cycle will occur, if the moisture threshold is not passed.

The water is being applied through an adjustable dripper mounted on the actuation component. Its supply is fixed at 25 L/h and the applications can be targeted as long as they are being carried out from the appropriate height. The – specific for each plant – dose is calculated based on the area of its bounding box. Five different classes and respective doses have been set (Low, Medium Low, Medium, Medium High, High) according to the literature [63,64,65]. The irrigation dose is expressed in seconds of pump operation. So, the respective dosages are 40 mL, 60 mL, 80 mL, 100 mL and 120 mL of water per lettuce plant that are applied to 6–17 sec of pump operation correspondingly. The aforementioned dosages are a subject of ongoing research. Based on the same classification method, the estimation of each plant’s height is derived. This estimate serves to slightly differentiate the height from which the irrigation is applied on each plant, indicatively a lettuce plant can reach 15–30 cm height.

Having processed the input data and given that the available soil moisture is deemed insufficient; the system will proceed to generate the commands for the robot’s actions.

#### 2.2.3. Generation of the Commands to Be Executed

This step combines all previous data and expresses it in a readable way for the GRBL software [66], a modification of which is installed into the Arduino microcontroller to control the stepper motor drivers and the irrigation component. GRBL is an open-source motion control software for CNC (Computer Numerical Control) milling that reads G-code (or RS-274) language, which is the most widely used CNC programming language. It is mainly used in constructions made with the use of computers for the control of automated machine tools, and has many variations.

**Figure 14:**
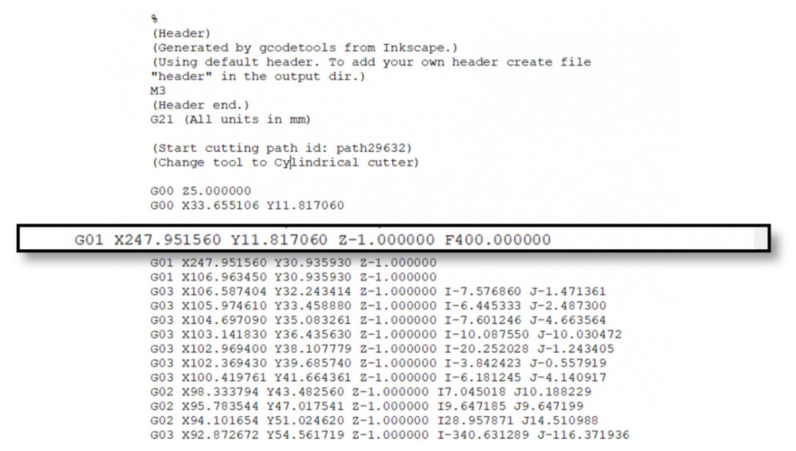
Example of a G-code section.

A G-code file may seem complicated at first but it is simple to understand. Almost every row has the same structure, and apparent complexity is introduced by numbers that are primarily Cartesian coordinates. An example is given in Figure 14.

In the selected row of the above code from a CNC milling machine can be observed the following:

- G##: G-code command, G01 translates as “move in straight line to a specific position”;
- X## Y## Z##: Cartesian coordinates X, Y, Z of the selected position;
- F##: Feed rate or speed at which the move will be executed [67].

In the example above more commands are observed, G02 translates as a circular motion from the previous point to the point X## Υ## Ζ##, with center of rotation the point I## J## and direction of rotation clockwise. Such commands, although necessary for a CNC system, had no utility in the present robotic system where it was deemed sufficient for all movements to be done linearly. Respectively, there is a very high accuracy in the motion coordinates; in the robotic system of the present work all movement commands can be and are executed successfully with an accuracy of 1 mm.

GRBL runs the produced code sequentially, receiving feedback for the successful execution of the previous sequence before executing the next. Having expressed, therefore, the required actions for the irrigation with G-code commands, the system will initiate the communication with the GRBL software installed in the Arduino to carry out the application. It shall be noted that during the image acquisition process at an earlier stage, the movement is executed in a similar way but for a fixed G-code file with given coordinates, as the points that the 5 images are captured are predefined.

#### 2.2.4. Movement and Application

Although there are variations between the application cycles, the general principle is the same. Initially, the system receives feedback on its position and confirms its starting position (X0, Y0, Z0—home). Before heading to the nearest as for the X axis plant, the vertical component (Z axis) is raised by 35 cm, which is set as the safe movement height for lettuce plants. Then, after the movement of X and Y axes, the actuator is placed above the first plant-target to be irrigated, descending to the application height set for the specific plant. The pump is then activated and runs until the defined dose for the specific plant is applied. Then, it gets switched off and the actuation component will be raised to the safe movement height. Axes X and Y moves the actuator above the next plant and the process is repeated for all identified plants. At the end of the cycle, the 3 axes will return to home and the robot will be switched off. In the next day the system will “wake up” again to follow the same processes’ sequence as shown in the flow chart (Figure 10).

## 3. Results and Discussion

The main experiments that were carried out and in particular those to extract the necessary metrics for their evaluation concerned: (a) the success of the image stitching process, (b) the realization of the plant identification and localization and (c) the precision of the robot’s movement. Each of the following subsections showcases the conducted experiments, the results and the discussion for each of the aforementioned aspects.

### 3.1. Image Stitching

**Figure 15:**
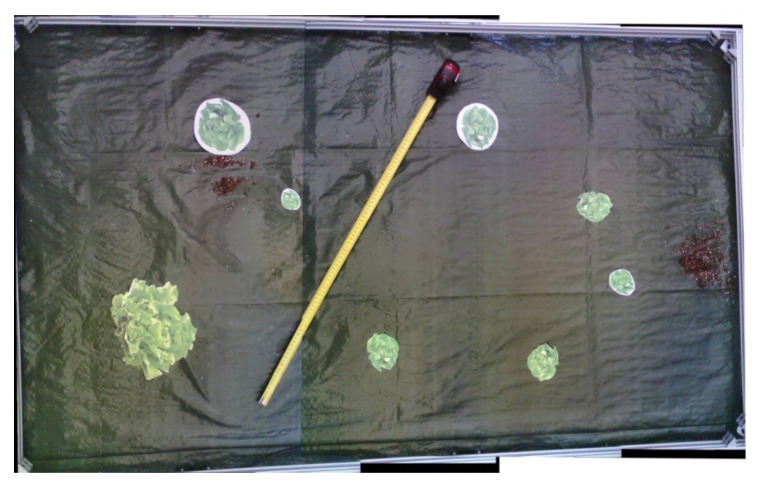
Example of a stitched image.

For the image stitching process even after selecting the OpenCV library, many different methods were tested mostly differentiated by the overlapping rate between 2 adjacent photos. The final method as presented in Section 2.2.2 was initially tested on a set of 6 equally divided—in 5 adequately overlapping final image sections—images. Three of them were manually cropped into 5 sections while the rest were real captures of the robot’s camera. The resolutions of the manually cropped images were similar to the camera’s captures (1600 × 1200 pixels). Figure 11 that was presented in Section 2.2.1 constitutes an example of the pictures that were stitched in the context of the experiment. In Figure 15 can also be seen an example of a stitched image out of 5 captures of the robot camera.

A segmentation of one of the parallel rails was observed at the lower side of the image. That segmentation was a result of the not absolutely vertical position of the camera’s mount. Even though this distortion slightly affected the efficient operation of the system, the camera mount was properly re-adjusted (Figure 11) and from that point forward it was realised that a continuous inspection of the camera’s vertical position was needed. The problem was later overcome with the optimization of the camera’s mount. In terms of software, the final stitching method lacks the issues arisen in previous implementations such as stitching inability or high distortion on the produced images, and works effectively, successfully combining the given images each time. 

As in Dissanayake V. et al. study [68], a visual evaluation and similarity comparison [69] with the real state of the garden parcel was carried out. The final validation was obtained when CityVeg was tested as a whole and was able to precisely move above each one of the plants (see Section 3.3. Precision of robot’s movement) and apply irrigation accurately.

### 3.2. Plant Identification and Localization

**Figure 16:**
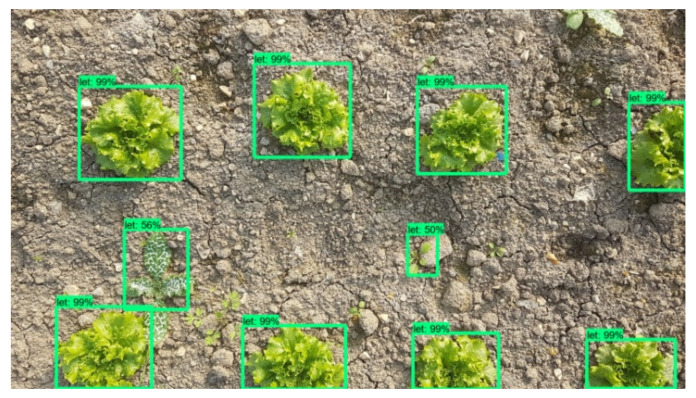
Plant detection and localization example.

As mentioned previously, the Faster-RCNN-Inception-V2 model was trained with 400 images containing 8 to 22 lettuces each, from early to advanced development stages. The evaluation of the model was carried out for 10 images, containing both lettuces and weeds. The point of view was similar to the images captured from the robot’s camera (top view) and the resolution was equal to the final combined image resulting from the stitching process. An example is given in Figure 16. In this particular image it can be observed that the model successfully detected all the lettuce plants and also detected and classified as lettuces 2 weeds, but with very low score (56% and 50%). In Table 1 the results from the 10 tests are presented in detail.

The detection task is faced as a classification problem between detected objects, in this case plants in bounding boxes, that are considered as lettuces; and undetected objects. In binary classification each input sample is assigned to one of two classes. In this case, the 2 classes are lettuces and non-lettuces. The labels that are assigned to these 2 classes are Positive for lettuces and Negative for non-lettuces. The metric values calculated for this task are Precision, Recall, Accuracy and F1-score. Although, the cost [70] of having a misclassified Actual Positive (or False Negative) lettuces is not very high regarding CityVeg operation, it is deemed necessary to present all the 4 metric values, in order to have an overall view of the system’s performance. A Confusion Matrix (Table 2) is used to visualize the cases in which the model struggles to discriminate between the 2 classes.

The 4 elements of the Matrix represent the following 4 metrics:

- True Positives (TP): Valid detections;
- False Positives (FP): Invalid detections;
- False Negatives (FN): Ground truths that are not detected at all;
- True Negatives (TN): Expected misdetections in the images, e.g., weed plants.

Deriving from Table 2, the 4 metric values typically used to evaluate object detection tasks are presented in Table 3.

Regarding the 4 metric values in the specific case of CityVeg, Recall indicates its ability to detect every single ground truth. When there is a high Recall score, it means that almost all ground truths (lettuces) will be successfully detected by the model. Precision answers the question of how many of the Predicted Positives are Actual Positives. It helps to quantify the confidence that a predicted bounding box is a valid detection indeed. Accuracy generally describes how the model performs across all classes. It is the ratio between the numbers of all correct predictions to the total number of predictions. F1-score is a function of Precision and Recall, as it is the Harmonic Mean of them and is a way to obtain a balanced feedback from both Precision and Recall scores.

The 4 obtained metric values are affected by an existing difficulty to identify the plants in early growth stages. So, at this point it is important present the metric values in respect to plants’ growth stage too. As it can be seen in Table 4, the scores achieved when early stage lettuces are about to be detected are significantly lower compared to those in advanced growth stage.

**Figure 17:**
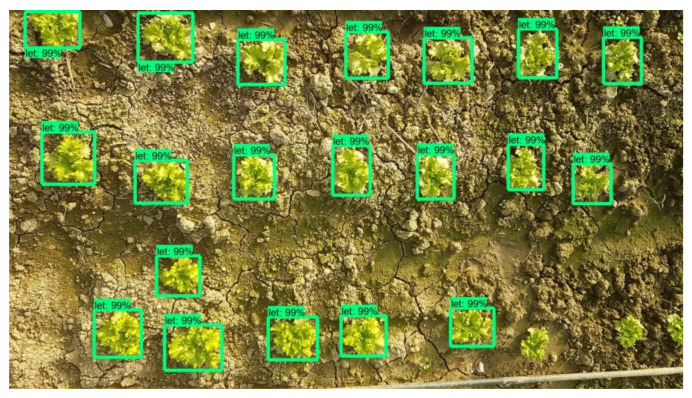
Plant detection and localization example in lettuce plants of early growth stages. CityVeg was unable to detect the bottom right lettuces which are the smallest ones in the picture.

**Figure 18:**
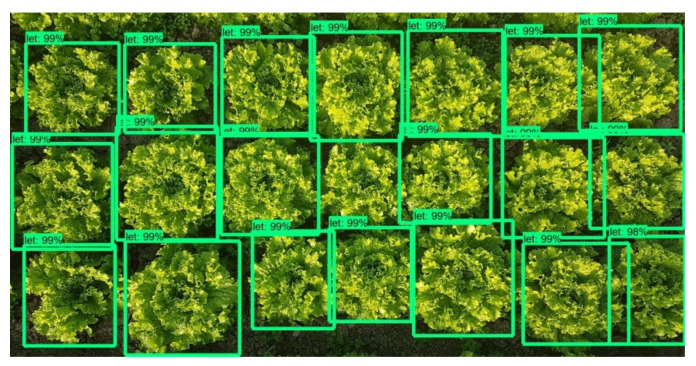
Plant detection and localization example in lettuce plants of advanced growth stages. All lettuces are successfully detected.

The reason for this is that from a growth stage and onwards, a lettuce plant has the same morphological characteristics and simply grows in size. Figure 17 and Figure 18 are indicative of the above fact. Faster-RCNN algorithm is able to recognize the patterns on which it has been trained, but the smaller ones have not yet formulated a clear leaf pattern. This is a common problem in plant detection tasks. Especially when the image resolution is not very high, the capacity of a Faster-RCNN algorithm to identify local texture is weak [71]. Several trials have been conducted and in recent years, an improved Faster-RCNN has been developed for this purpose. Relatively large errors when detecting the leaf area of very small lettuce plants are observed as well by Lu et al., even when using Mask-RCNN algorithms [72].

In recent years, researchers have achieved good results in small object detection in optical remote sensing images through the improved Faster R-CNN [13].

According to the above results, the model is able to detect the majority of lettuce plants. The difficulty faced regarding smaller plants at this stage of development is not significant, but at a later stage of CityVeg’s development it could become a major problem, e.g., weeding on the wrong target. A possible solution would be for the model to be further trained with more plant images at earlier development stages. A possible replacement of the installed camera will be considered by the time weeding function is added, as the low score can also occur due to its generally low resolution (low cost camera).

It is very promising that mature plants are successfully identified as separate plants even in cases where there is overlap between the foliage of nearby plants. In a few cases where identification of lettuce plants in advanced growth stage failed, it was most likely due to the absence of a complete view of the plant. In the demarcated garden parcel of the system no such issue arises.

Additionally, weeds are sometimes identified as lettuce plants with a low score (False Positives). False Positive means that a non-lettuce plant has been identified as lettuce. When the cost of False Positive is of high importance, Precision is a necessary metric to determine. However, in the application of CityVeg, if the Precision score is not high for the lettuce detection model, it will not constitute a major problem for the time being. Identifying weeds as desirable plants will be a problem in the future steps of the robotic system; as it should not boost the weeds by irrigating them.

On the other hand, if, in example, an actual lettuce is not predicted as lettuce, it is a False Negative and is not detected at all. The cost of not detecting some existing lettuces is also not so important for CityVeg. The reason for this is that it is quite possible to detect them during the forthcoming days, since as lettuces grow bigger; it is easier for the model to detect them. Apart from that, the soil moisture of a properly irrigated garden should be enough to maintain them, even not under ideal watering conditions, until they can be detected. As previously, it is considered advisable to further train the model with additional lettuce plants in early development stages.

### 3.3. Precision of Robot’s Movement

**Figure 19:**
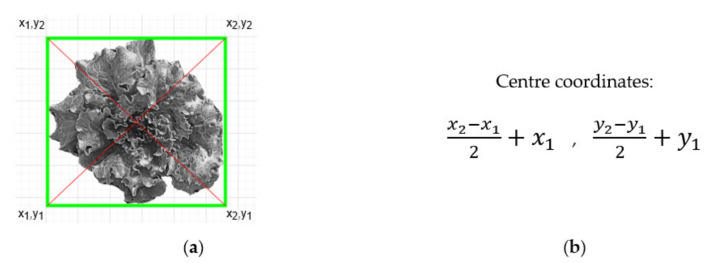
(**a**) Schematic representation of the exporting of the bounding boxes’ centers and; (**b**) Equation used to extract the centre’s coordinates.

As mentioned before, after the detection process, the model exports data from the bounding boxes enclosing each plant. Boxes’ angles are utilized to export their centres (Figure 19a) and their coordinates (Figure 19b). These centres are the “targets” that the actuation component must navigate to in the highest precision possible, so to circularly apply irrigation around them through its dripper. Due to the type of the dripper, the area covered is large enough and no particular precision is pursued. However, when weeding function is added, the precision movement requirements will be high as the pesticide efficacy will be positively affected by an improved, i.e., millimeter-level, deposition accuracy. The aim was to ensure the latter by this development stage already. Therefore, the requirements for CityVeg’s positioning accuracy can be summed up to: (a) precisely moving above each plant, (b) being able to repeat, (c) repeating the movement for as high number of repetitions as possible, without accumulating errors.

**Figure 20:**
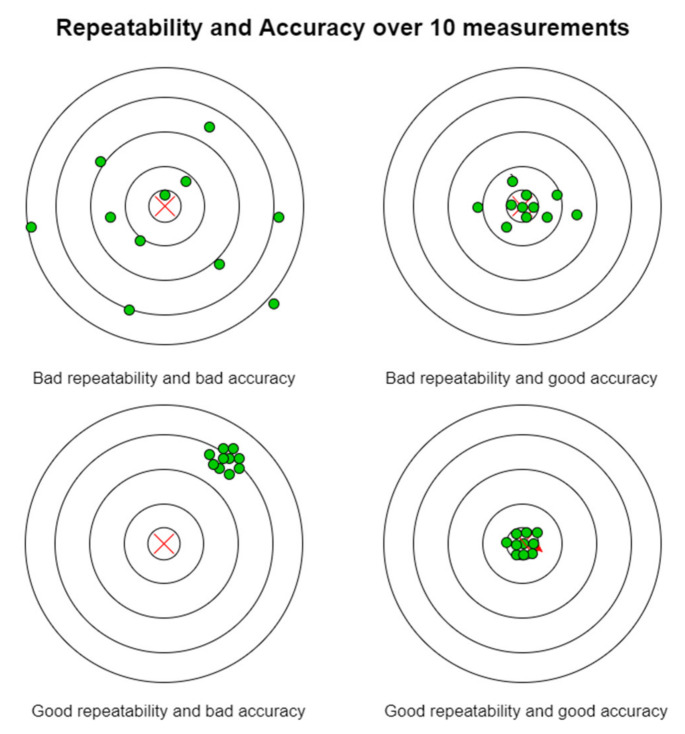
Repeatability and accuracy after 10 measurements. Adapted from [73].

The characteristics of CityVeg that were measured in this experiment were its repeatability and its accuracy. Repeatability can be briefly defined as its ability to repeat the same task while accuracy is the difference (error) between the requested and the finally obtained task. Thus, in this system repeatability refers to the movement at the same point over and over, while accuracy refers to finding the correct plant/target each time (Figure 20).

Regarding the precision that the actuator reaches each particular plant (target), it has to be clear that at this point of the CityVeg’s main function—that is irrigation—although it is desirable to achieve high accuracy in its movements, there is no need for accuracy in millimetres level. Undoubtedly, this kind of applications can be approached with some tolerance. However, CityVeg is designed from scratch to be efficient and perform minimum to zero deviations (as much as possible). In this context, there are 3 main factors that ensure that CityVeg accurately approaches the targets:

1. The selection of stepper motors: A great characteristic of stepper motors is their ability to position very accurately, as they rotate in discrete steps. Wantai Motors’ bipolar stepper motors that are used in CityVeg [74] have 200 steps per revolution which is translated to 1.8° rotary movement per step. According to their specifications, there is a possibility for some little inaccuracies as they have a tolerance of ±5% error regarding the location of any given step. This means that any step on a typical 200 step per revolution motor will be within a 1.8° error range. However, the special advantage that stepper motors provide is that the possible error does not accumulate from step to step. More specifically if a stepper motor travels one step, it will turn 1.8° ± 0.09°. However, if the motor travels one million steps, it will travel 1,800,000° ± 0.09°. The angle between two consecutive steps N and N+1 may have up to 5% error, but since the sum of the angles between each step add up to 360°, the motor’s rotor is going to be back to where it had started from after 200 steps. In applications that high accuracy movement is really important, standard 200 step/rev stepper motors can be geared down by more than 10:1 and use full or half steps only, i.e., gearhead stepper motors. Furthermore, an auxiliary technique can be used for noise and vibration reduction also increasing effective torque, by reducing mid-band resonance effects. Steppers can be driven by a very useful controlling capability called micro-stepping [75]. Adjusting properly the drivers which send the signals (pulses) into the motor causing it to turn, the degrees of the rotary movements can be reduced by factors of 2. For example, in case of 1/16th micro-step setting, that would give an angle of 0.1125° per step. During micro-stepping, it is not necessarily the accuracy that increases, but the resolution.
2. Motor settings: Regarding the settings, two factors were taken into account even from the initial design stage. First, the steppers were carefully selected to be able to serve more than their rated load and the provided detent torque of 2.6 N∙cm ensures that such problems will not arise. Second, aiming to perform a smooth motion operation, the Π frame’s maximum speed has been set to 800 mm/min, and the vertical components to 350 mm/min, which are rather low. Although, there is room to increase the speed, this is not necessary at all as it doesn’t make any change if the operation is completed faster.
3. Detailed construction and selected materials: The whole construction is assembled tightly in a way that both the rectangular frame and the Π frame are stable enough and compact, avoiding major oscillations. As it was explained in Section 2.1.1, the aluminium V-slot linear rails and plates are tightly screwed together with joints. Regarding the transmission, the neoprene and fibreglass cords made of timing belts are properly tightened and frequently checked if adjustments are needed. Another important characteristic is the size of CityVeg construction, which is tailored to serve an area of 1.75 cm^2^ (XY plane). In case that the size was e.g., 5 times greater than the existing one, it is not sure if the designed construction could provide adequate stability or if the movement accuracy would be affected. Further experiments and testing would be necessary to explore if the proposed design can be scaled up.

**Figure 21:**
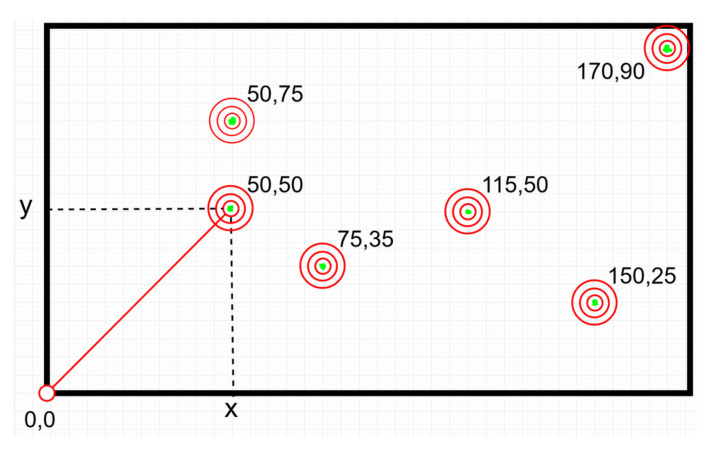
2D schematic representation of the set paths. The green dots represent the pen marks. In some cases the dots are less than 10 due to overlap between them.

In order to measure how precise the movements of the robotic system are, 2 different tasks were carried out. The first task is about checking if the actuator reaches every target with adequate accuracy. Initially, 6 targets were set on the garden area. The positions of the 6 targets were manually marked on a millimetre paper on the floor, in respect to the “home” position. To perform the measurements the actuator was removed and a pen was mounted instead. Considering as axis origin (“home”) one of the corners of the aluminum frame, tests were conducted for 10 iterations. The actuator moved from “home” position to each of the 6 targets consecutively, simulating a normal irrigation task for a 10-day routine. The performance of the robotic system was recorded in terms of the distance between the coordinates of the actuator and each target center point (Figure 21). The results obtained are presented on Table 5.

Based on the above table the mean for all iterations for the movement to target (150, 25) is 0.4 mm, indicating the distance from the real target (accuracy); while the standard deviation for the same point is 0.49 mm, indicating that all iterations reach up to that far from the desired target (repeatability). The respective mean for (170, 90) is 0.8 mm, while the standard deviation is 0.4 mm. All the other targets were approached without any deviations from the center points (zero values for both mean and standard deviation respectively).

The Z axis’ movement accuracy was not evaluated in this experiment, as it was only slightly moved up and down for the experiment’s purpose (marking). It shall be noted that (a) this experiment was performed before the installation of the limit switches that improve the system’s movement accuracy by reducing any cumulative error since in each cycle the system “resets”, and (b) it was the final of a series of experiments aiming to optimize the—absolute—movement accuracy. In the 10 operating cycles the pen’s point each time almost coincided with the set one (deviation of millimeter level due to the diameter of the manual mark).

**Figure 22:**
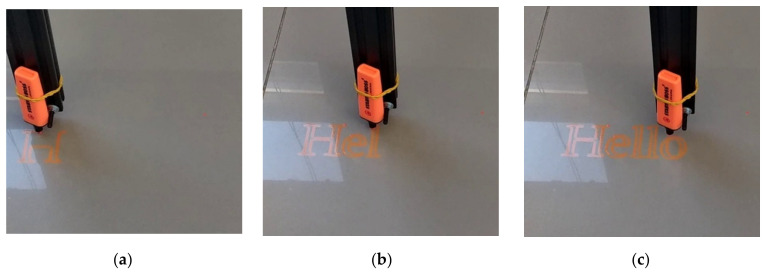
CityVeg second movement test, “Hello”. (**a**) Step 1/3, (**b**) Step 2/3, (**c**) Step 3/3.

During the second test, the intention was to ask from CityVeg to perform a much more complicated task than the ones that it is designed to carry out, such as the previous test case. Looking at CityVeg as a CNC (Computerized Numerical Control) machine or 3D printer, an orange marker was mounted on the actuator position and a file/script containing the exact spots of a drawing was given to CityVeg as input (Figure 22).

Judging by the final result, CityVeg was capable of depicting an extremely realistic floor drawing of the provided script. This visual validation indicates that there are no significant deviations from the targets that CityVeg is asked to approach. Both test results are considered sufficient for the performed application of irrigation, as this level of inaccuracies does not affect the performance of CityVeg in real world applications.

There is a possibility that the reason for the performed inaccuracies is that one stepper motor has missed a step. When this happens, a motor that is being at a position N, fails to move to position N+1 after the given command (pulse). This is usually derived either from (a) the load on the motor when it is too large and the motor doesn’t have enough torque to move to the next step with the load, or (b) the preselected speed of the motor when it is too high and causes the motor to step too fast.

In our case, the results indicate accurate movement as both (a) and (b) factors have been taken into consideration. However, when inaccuracies are very frequent and increasing over time, a series of interventions can take place to further optimize the accuracy. When there is need to detect if the used stepper motor has missed a step, an optical encoder can be mounted on the motor, which will determine the exact position of the shaft. In addition, a gearbox could be incorporated to the motor’s shaft. In cases where the problem of inaccuracy is of much importance, it has also to be taken into account that the accuracy of a motor is directly dependent on the step size of the motor itself. Considering this, it may be necessary to replace the existing stepper motors. For example, a 0.9° step angle motor is more accurate than a 1.8° one as, from a mechanical point of view, there are more steps introduced. More specifically, for 0.9° step angle motors 400 steps can be achieved while for 1.8° step angle motors 200 steps can be achieved, per revolution. The difference lies on the rotor design of 1.8° and 0.9° motors, as it can be seen that a 1.8° motor has 50 teeth whereas a 0.9° step motor has 100 teeth. The aforementioned property results to higher torque stiffness and thus, higher accuracy.

According to the test results and the overall performance of the robot, it was not deemed necessary to apply any of the available corrective interventions, as the achieved movement accuracy is adequate for the purpose of irrigation.

To the existing accuracy level is added the extra safety factor provided by the presence of limit switches, which contribute to the correct finding of the “home” position before each cycle. There were minor deviations between the navigation of the actuation component to: (a) the centre of each bounding box containing a plant and (b) to the actual centre of each plant, but finally these deviations are not manageable with the selected classifier. As explained, much of the accuracy in movement is due to the stepper motors and their driving software. However, the quality of the individual components of CityVeg also contributes to this. The selection of pulleys, the adjustment of the distance between the wagons’ wheels, the securing of the belts and the addition of belt tensioners and more, all contribute to the system being able to perform many repetitions without constantly requiring adjustments due to wear. Of course, as a mechanical structure with moving parts, maintenance will be required from time to time.

Implementation of CNC movement techniques in agricultural tasks as presented in similar research works published [76,77]; prove that by following the concept of Cartesian robots the issue of precision movement is approached either by setting predefined coordinates, or by detecting the desirable targets using other detection algorithms as YOLOv3. Compared to the solutions that use predefined, given spaces or plant spots, the developed in this work robot, does not rely on fixed positions, but scans the garden parcel and detects/localizes the plants itself. This allows the user to freely plant wherever he prefers, and the robot will adapt. This is something that allows for a better utilization of the usually confined space of an urban garden parcel. Apart from that, the irrigation dose is not a fixed curve depending on the days after seeding only, but is daily updated taking into consideration the detected area (top view) of the plants too. This dynamic aspect ensures the continuous fitting of the dose. Especially when the NDVI (Normalized Difference Vegetation Index) plants’ values will be obtained, a further, more qualitative assessment of the plants’ status will be achieved and the dose will be even better adapted.

#### Hardware Issues Regarding Robot’s Movement

After the design, during the process of construction, programming and testing of CityVeg a number of simple or more complex problems arose and were addressed, presented below.

One of the first problems that arose during the construction of the robotic platform was the stability of the Π frame on the two horizontal rails. The placement of the rectangular wagons (127 mm × 88 mm × 3 mm) could be done either horizontally or perpendicular to them. The horizontal layout that was initially tested had the advantage of greater wheelbase and therefore greater stability against frame oscillation. This arrangement, however, did not allow the vertical supports of the vertical sections of the Π frame to be supported on the two wagons. For that reason the vertical arrangement shown in Figure 4b was preferred; and sufficient stability was ensured thanks to the eccentric spacers of the upper wheels of each wagon. It shall be noted that due to the weight carried in the upper, horizontal part of the Π frame, when a movement of the X axis comes to a halt, and especially when the vertical bar - axis Z - is far from the middle of its available movement length, a very short oscillation of the frame is observed. In the process of capturing 5 images for creating the garden parcel panorama, this is encountered with an additional 1.5 s halt prior to each shot.

Another issue that arose during the construction of the robot was the support on the third wagon of all the necessary components: 3rd wagon’s wheels, vertical bar’s support wheels, vertical bar’s drive motor, camera. The solution was provided by the especially designed for the work’s needs camera and motor bases, which were 3D printed.

Other important problem consisted the interferences in the stepper motors when the pump was activated, which resulted in their spasmodic movement but also the noise in the interfaces of the terminal switches. These problems have been successfully addressed thanks to the proper rearrangement of ground connections for pump interference, and the proper combination of resistors and storage elements (capacitors) between the terminal switches and their microcontroller.

Finally, the order of irrigation of each plant was an issue to be resolved. Plants identified by the Faster-RCNN are placed in an unordered list. Their classification will also define the path which the actuator will follow in order to apply irrigation in each individual plant. In a system like this, there is no need for fast execution of processes, but a correct classification method that would lead to the shortest possible path, would contribute to less consumed power and in the long run, to less system stress. In the end, the movement of the actuator was chosen to be based on ascending X coordinates, a choice which largely serves the smooth movement of the robot.

## 4. Conclusions

The experimental procedure proved that the developed robotic system adequately performs all of the aforementioned processes in lab scale. According to the system pipeline, the developed algorithm acquires the garden data as input, processes it and exports the information needed in order for the robot to precisely irrigate the desired plants according to their specific needs. The precise spatial navigation, meaning the detection, approaching and application upon the detected plant, is a direct result of the successful operations of depiction of the garden parcel and processing them to extract the real scale, through the obtained images. As mentioned previously, all training and experiments were carried out for lettuce plants. It can be observed that CityVeg successfully detects the mature plants, but is not yet ready to detect all the very small ones. This is going to be a forthcoming research topic to be explored and the steps towards this are: (a) the enrichment of the dataset with more plants of early growth stage and multiple weeds, (b) the testing of a new camera with a higher provided image resolution and (c) the installation of a LED flash light on the bottom of the Π frame that will ensure standard lighting conditions during the process of image acquisition. The promising results of image stitching, plant detection and localization and movement precision allow the further development and optimization of the system with the future steps presented next.

After the system is tested in real conditions for the specific crop and the necessary adjustments in its functional aspects are made, the next steps will prioritize in: (a) addition of fertigation (utilizing Normalized Difference Vegetation Index (NDVI)), (b) weeding capability, making the proper modifications in system’s hardware and software, (c) optimization of the applied dosages for each type of application with tests in real conditions, (d) training of the whole algorithm in order to support more plants, (e) addition of more sensor types in order to holistically monitor the urban garden, e.g., air humidity/temperature, light intensity, etc., and (f) development of a mobile App for remote monitoring and record keeping.

## Figures and Tables

**Table 1:** Plant detection test results.

No. of Image	No. of Lettuce Plants in the Picture	No. of Detected Lettuce Plants	Comments
1	8	8	Plants at early stage of growth, 2 weeds detected as lettuces (FP) but with a low score.
2	17	15	Plants at advanced stage of growth with partial overlap of the foliage.
3	17	16	Plants at advanced stage of growth, bounding box incorrectly placed in 1/17 plants and two nearby plants were detected as one.
4	16	15	Plants at advanced stage of growth.
5	17	16	Plants at advanced stage of growth with partial overlap of the foliage. Two nearby plants were detected as one.
6	21	21	Plants at advanced stage of growth.
7	11	10	Plants at early stage of growth, a small lettuce was missed (FN).
8	13	14	Plants at early stage of growth. Two nearby plants were classified as individual plants with very low scores (42% and 47%) and as a single plant with a high score (94%) (FP).
9	15	14	Plants at early stage of growth, 1 small lettuce was missed (FN).
10	22	19	Plants at early stage of growth, 3 small lettuces were missed (FN).

**Table 2:** Confusion Matrix.

	Actually Positive	Actually Negative
Predicted Positive	148 (TP)	4 (FP)
Predicted Negative	9 (FN)	2 (TN)

**Table 3:** Recall, Precision, Accuracy and F1-score for all lettuce plants.

Metric Value	Recall	Precision	Accuracy	F1-Score
Score	0.9426	0.9736	0.9202	0.9579

**Table 4:** Recall, Precision, Accuracy and F1-score separately for lettuce plants at early growth stages and advanced ones.

Metric Value	Recall	Precision	Accuracy	F1-Score
Early growth stages	0.9375	0.9615	0.9059	0.9494
Advanced growth stages	0.9481	0.9865	0.9359	0.9669

**Table 5:** Repeatability and accuracy test results.

Target	Coordinates (X, Y)	Distance in mm from the Target’s Center (mm)									
		Iterations									
		1st	2nd	3rd	4th	5th	6th	7th	8th	9th	10th
1	(50, 50)	0	0	0	0	0	0	0	0	0	0
2	(50, 75)	0	0	0	0	0	0	0	0	0	0
3	(75, 35)	0	0	0	0	0	0	0	0	0	0
4	(115, 50)	0	0	0	0	0	0	0	0	0	0
5	(150, 25)	0	0	1	0	0	1	1	0	1	0
6	(170, 90)	0	0	1	1	1	1	1	1	1	1

## Appendix A

**Table A1:** List of components.

	Component	Short Usage Explanation	Quantity
1	V-Slot 2040 1500 mm—Natural Anodized	Parallel horizontal bars	2
2	V-Slot 2040 1000 mm—Natural Anodized	Parallel horizontal bars stabilization	3
3	V-Slot 2040 250 mm—Natural Anodized	Various extensions	5
4	V-Slot 2020 1000 mm—Natural Anodized	Vertical bars of Π frame	2
5	V-Slot 2040 1000 mm—Black Anodized	Vertical bar	1
6	V-Slot 2040 250 mm—Black Anodized	Vertical bar extension	1
7	V-Slot Gantry Set 2020 × 2080 Xtreme	X, Y axes wagons	3
8	Inside Hidden Corner Bracket 2020	-	12
9	Cast—90 Degree Corner Bracket	-	40
10	Stepper Motor Mount Plastic NEMA 17	-	1
11	Ζ axis motor base—3D printed	-	1
12	Stepper Motor Mount—NEMA 17	X, Y axes motor mounts	3
13	Idler Pulley Plate	X, Y axes idler pulley plates	3
14	Timing Belt—GT2	-	11 m
15	Belt Locking—for GT2 Belts	Belt tensioners	3
16	Aluminum GT2 Timing Pulley—6 mm Belt—20 Tooth—5 mm Bore	Toothed pulleys	3
17	OpenBuilds Smooth Idler Kit	Free pulleys (opposite side than motors)	3
18	Stepper Motor 2.8kg.cm (200 steps/rev) 42BYGHW208	-	4
19	OpenBuilds Solid V Wheel Kit	Vertical bar support/scroll wheels	5
20	Eccentric Spacer—6 mm	Eccentric spacers for vertical bar’s wheels	6
21	Spacer Sleeve ID 3.6 mm L 5 mm—Black	Spacers for Z axis motor support	6
22	Z axis motor gear (D-shaft)—3D printed	-	1
23	Rack Mod.1 15 × 15 mm split in 18 cm individual parts—3D printed	-	6
24	Quad Tee Nut—MAKERLINK	120^ο^ frame’s quad connecting Tee Nuts for end to end V-Slot connections	20
26	OpenBuilds Tee Nuts M5	Nuts—V-Slot	75
27	Bolts M5—L 10 mm Low Profile Black	Frame support screws—V-Slot	75
28	Endcap for 2040 V-Slot—Green	-	2
29	Endcap for 2020 V-Slot—Green	-	2
30	Rubber Cap/Foot (Rubber only)	Temporary rubber feet	8
31	Power Supply Industrial 12 V 8.5 A 102 W MeanWell—LRS-100-12	-	1
32	Arduino Mega2560 Rev3	-	1
33	A4988 Stepper Motor Driver	-	4
34	OpenBuilds Micro Limit Switch Kit with Mounting Plate	Limit switches’ kits	8
35	Bluetooth Module for Arduino—HC05	Communication tests	1
36	Camera Module based on ESP32	-	1
37	Camera housing and base—3D printed	-	1
38	Soil Hygrometer Module	-	1
39	NodeMCU—Lua based ESP8266	-	1
40	ESP8266 WiFi Module ESP-12	-	1
41	DC-DC Step-Down 1.3–35 V 3 A	-	3
42	Liquid Pump Motor—Micro 12 V	-	1
43	Polyethylene irrigation pipe of 6 mm	-	8 m
44	Dripper 14–70 L/h	-	1
45	Cable Drag chain 10 × 10 mm 1 m	Cable management	2
46	Construction box 176 × 126 × 57 mm	Electronic equipment protection	1
47	Other components such as:	Screws, washers, nuts, cables, cable ties, heat-shrinkable cable insulation, resistors, capacitors, etc.	-
